# Comparison of Two Metabolic Simulators Used for Gas Exchange Verification in Cardiopulmonary Exercise Test Carts

**DOI:** 10.3389/fphys.2021.667386

**Published:** 2021-06-03

**Authors:** Tjeu Souren, Edward Rose, Herman Groepenhoff

**Affiliations:** ^1^Independent Consultant, Utrecht, Netherlands; ^2^Independent Consultant, Novi, MI, United States; ^3^Vyaire Medical, Mettawa, IL, United States

**Keywords:** cardiopulmonary exercise test, metabolic simulator, respiratory exchange ratio, calibration, metabolic cart, Vyntus ONE, Vyntus CPX

## Abstract

**Introduction:**

Metabolic simulators (MS) produce simulated human breaths for the purpose of verification of cardiopulmonary exercise test (CPET) equipment. MS should produce consistent identical breaths with known CO_2_ and O_2_ gas concentrations over a range of breath rates and tidal volumes. Reliability of a CPET metabolic cart depends on ongoing quality control and maintenance of the device, including intermittent verification with a MS. We compared two MS devices against two standard CPET systems.

**Methods:**

The Vacumed 17056 (Vacumetrics, Ventura, CA) and Relitech (Relitech Systems BV, Nijkerk, The Netherlands) were used with two standard metabolic carts (Vyntus CPX and Vyntus ONE, both Vyaire Medical, Mettawa, IL, United States). Tidal volume (VT) was set at 2 and 3 L and breathing frequency ranged from 20 to 80 breaths per minute for each MS. At each set point, we measured three sets of 40 breaths. Primary outcome parameters collected were VT, oxygen consumption ($\dot{v}$O_2_), carbon dioxide production ($\dot{v}$CO_2_), and respiratory exchange ratio (RER).

**Results:**

VT, RER, $\dot{v}$O_2_, and $\dot{v}$CO_2_ results as obtained from both MS were all within the limits of acceptability, at both tidal volume settings, and all ventilatory rates. No significant trends were identified for either MS device. The Relitech MS produced tidal volumes that were closer to the target VT for both CPET carts at both VT and all rates, but the results of both MS were within acceptable ranges.

**Conclusion:**

Verification of CPET equipment using either the VM or RT metabolic simulator, producing highly accurate and predictable simulated breaths of known composition, enabling CPET laboratory managers to rely on subject test data obtained during cardiopulmonary exercise testing.

## Introduction

Cardiopulmonary exercise testing (CPET) is increasingly used for diagnosing complex medical conditions covering a broad spectrum of conditions from cardiopulmonary diseases to metabolic and even mitochondrial disorders (Muraresku et al., 2018). CPET is helpful for identifying the presence and severity of heart failure, atherosclerotic heart disease, peripheral vascular disease, pulmonary hypertension, deconditioning, restrictive, or obstructive pulmonary disease, alveolar diffusion disorders, and cardiomyopathy (American Thoracic Society, 2003). CPET is also widely used for tracking progress of performance testing, rehabilitation and training, response to treatment, risk assessment for major surgery, and eligibility for transplant (Levett et al., 2018). Results from CPET can guide nutrition and parenteral replacement therapy (West et al., 2017).

The data from CPET provide a highly accurate evaluation of linked physiological systems—ventilation, pulmonary gas exchange, circulation, and metabolic processes—against a known workload (American Thoracic Society, 2003). The ability to identify subtle changes in metabolic rate and metabolic efficiency at a tissue, cellular, or even organelle level requires highly sensitive and accurate equipment producing reliable data. To ensure reliability of CPET equipment, quality control should be performed on a routine basis. Society guidelines from the American College of Chest Physicians (ACCP), American Thoracic Society (ATS), and European Respiratory Society (ERS) as well as the manufacturers of the CPET metabolic carts recommend specific calibration procedures, combined with periodic external maintenance, calibration, and verification against known standards (American Thoracic Society, 2003). CPET laboratories must have confidence that the external systems used for calibration or verification are certified, accurate, and reliable “gold standards.” To date, research often compares CPET machines against a metabolic simulator (Casaburi et al., 1997; Porszasz et al., 2007; Beijst et al., 2013; Van Iterson et al., 2016; Levett et al., 2018; Radtke et al., 2019), although evidence that the metabolic simulators themselves are accurate and reproducible has been lacking (American Thoracic Society, 2003).

Automated breath and metabolism simulators, used for the validation of CPET systems, have been used for over 30 years. Simulators provide reproducible simulations of human breathing at various breathing frequencies and tidal breath volumes. Ideally, the respiratory and metabolic variables of tidal volume (VT), respiratory rate (RR), minute ventilation ($\dot{v}$E), oxygen consumption ($\dot{v}$O_2_), and carbon dioxide production ($\dot{v}$CO_2_) generated by a metabolic simulator should be indistinguishable from the patterns produced by humans. The simulator produces and controls those variables with high precision and consistency so that every breath, at every breath rate, is identical. Consequently, one can test any metabolic cart with high precision, reproducibility, and accuracy. The validity of VT, respiratory exchange ratio (RER), $\dot{v}$O_2_, and $\dot{v}$CO_2_ produced by a simulator is crucial (Casaburi et al., 1997; Gore et al., 1997).

The respiratory exchange ratio (RER) is one metabolic parameter reported by CPET carts. RER is defined as the ratio between the amount of exhaled $\dot{v}$CO_2_ from metabolism and $\dot{v}$O_2_ uptake, resulting in a dimensionless value that correlates with the metabolic rate (respiratory quotient) and the fuel being used by the subject (fats vs. carbohydrates). If the CPET laboratory staff follows the manufacturer’s recommended onboard calibration procedures and external validation, it is assumed that the metabolic analyzer will produce precise and valid test results (within the specifications of the manufacturer of the device) from which clinical decisions may be made. However, this may not always be true if there is discrepancy between metabolic simulators (Myers, 1996).

The operation of the metabolic simulator utilizes a high precision motor-driven cylindrical piston pump similar to a syringe that creates very precise stroke volumes of air (VT) at adjustable stroke rates (breathing frequency, BF). By injecting known amounts of CO_2_ and N_2_ into the stroke volume, an exact volume of CO_2_ and of O_2_ per breath is created. Nitrogen is infused to reduce the O_2_ concentration of the ambient air to simulate oxygen consumption, and CO_2_ is added to the ambient air to simulate CO_2_ production. The gases, CO_2_ and N_2_, are added to the simulator’s tidal strokes through highly precise mass flow controllers (Huszczuk et al., 1990). With the injection of known masses of CO_2_ and N_2_, in combination with adjustments for pump stroke frequency and tidal volume (VT), accurate $\dot{v}$O_2_, $\dot{v}$CO_2_, and $\dot{v}$E can be created.

Both Relitech and Vacumed metabolic simulators share the same design principles. The user interface allows the operator to set different stroke volumes (1–3 L, in steps of 0.5 L) and adjustable stroke frequency (from 10 to 80 strokes^∗^min^–1^), thus enabling simulated ventilation ranges from 10 L^∗^min^–1^ up to 240 L^∗^min^–1^ at a simulated breathing rate up to 80^∗^min^–1^. The metabolic gases for both systems are mixed by the metabolic simulator using room air that is pumped back and forth, injecting amounts of pure CO_2_ and N_2_. The 100% CO_2_ creates a gas that simulates a precise CO_2_ production rate at the different stroke rates (ventilation), while 100% N_2_ dilutes the ambient air O_2_ to a specific O_2_ concentration to simulate pre-set oxygen consumption rates. The amount of injected CO_2_ and N_2_ during each breath exhaled by the metabolic simulator is regulated by high precision mass flow controllers (MFC’s), resulting in a level of precision of simulated metabolic rate for O_2_ and CO_2_ of <0.2%. Certification of the metabolic simulator piston pump and MFC’s are performed initially, with re-certification every 2 years by the manufacturer of the MFCs. Together, this level of volume and MFC precision enable the metabolic simulators to create $\dot{v}$O_2_ and $\dot{v}$CO_2_ with an accuracy of <0.5% even at high ventilation ranges.

International guidelines recommend the use of a metabolic simulator to perform a thorough systematic check on the overall performance of a CPET metabolic system (Casaburi et al., 1997; Porszasz et al., 2007; Radtke et al., 2019). However, the reliability and comparability of different brands of commercially available metabolic simulators have not been discussed in the literature and may not necessarily be consistent across CPET carts (American Thoracic Society, 2003; Bunn et al., 2011). Therefore, we conducted a study to verify the uniformity of outcome response of two types of metabolic simulators against two standard CPET metabolic carts.

## Materials and Methods

**FIGURE 1:**
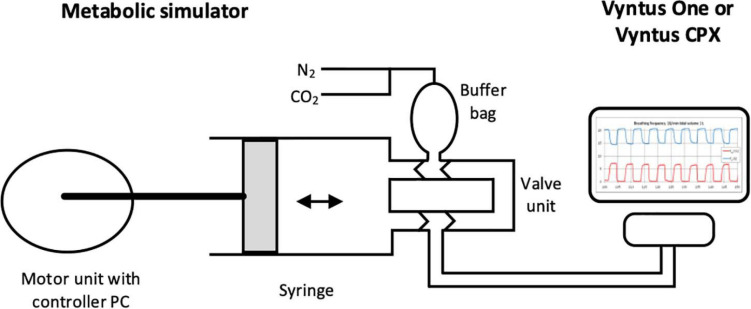
Schematic view of metabolic simulator with sample output (original artwork).

Two metabolic simulators equipped with mass flow controllers (Vacumed 17056, Vacumetrics, Ventura, CA, United States and Relitech, Relitech Systems BV, Nijkerk, The Netherlands) were used with two standard metabolic carts (Vyntus CPX and Vyntus ONE, both Vyaire Medical equipped with Sentry Suite Software version 3.10, Mettawa, IL, United States). The Vacumed (VM) metabolic simulator used for this investigation was modified to include two mass flow controllers (MFC), one for 100% oxygen and one for 100% nitrogen, instead of its typical one MFC for a gas mixture, to more closely mimic the double MFC setup in the Relitech (RT) system (see Figure 1).

The Vyntus CPX uses a calibration and verification procedure that is fully automatic using a built-in flow generator, producing precise constant airflows. This sets a flow calibration factor using a calibration flow at 2 L^∗^s^–1^ followed by a low flow verification at 0.2 L^∗^s^–1^ with limits of acceptability of ±1% of the expected flow. The 2 L^∗^s^–1^ flow is used to set the gain: the measured flow at 2 L^∗^s^–1^ should be within 1% of the expected flow, and a gain is set to adjust. The lower flow rate of 0.2 L^∗^s^–1^ is for verification of the digital (rotating vane) volume sensor for undisturbed rotations at flow initiation.

In contrast, although the Vyntus ONE uses the identical digital volume sensor, volume is manually calibrated using a certified 3 L calibration syringe. A manual verification procedure is performed, discharging the 3 L syringe at least three times to give volume strokes of 3 L at flow ranges from 0.5 up to 12 L^∗^s^–1^ (Porszasz et al., 2007). The limits of acceptability are 3 L ± 3.5% for each flow range, according to international standards (Miller, 2005).

Both Vyntus models use the same gas sensors: a high speed digital O_2_ sensor based on an electrochemical principle; and a fast response digital CO_2_ sensor based on the principle of infrared absorption. For both Vyntus models, the gas calibration procedures are the same, consisting of determining the O_2_ and CO_2_ signals, initially analyzing room ambient air and subsequently a highly accurate calibration gas mixture consisting of CO_2_, O_2_, and balanced nitrogen (nominal 5% CO_2_ and 15% O_2_ in N_2_, with 0.05% accuracy). The calibration certificate values as defined by the calibration gas supplier are set as reference values. The gas calibration procedure determines gain factors, response time, and delay time for the O_2_ as well as the CO_2_ sensor.

Both metabolic simulators were used in random sequence for both CPET devices. The primary CPET parameters (VT, RER, $\dot{v}$O_2_, and $\dot{v}$CO_2_) were verified using a standardized protocol: the testing sequence was first performed with a tidal volume of 2 L and subsequently 3 L at different frequencies: 20^∗^min^–1^, 40^∗^min^–1^, 60^∗^min^–1^, and 80^∗^min^–1^. Every frequency level consisted of three sets of at least 40 “breath” cycles. Vyntus CPX and the Vyntus ONE were both set to breath by breath (BxB) mode.

Metabolic simulators operate in ambient conditions, producing the pre-set breath cycles using ambient temperature and pressure conditions (ATP). Within the Vyntus system application software (SentrySuite^®^), a setting was used (SeS QM) which stops the conversion of “breaths” from ATP to body temperature, pressure and water vapor saturated (BTPS) for VT and standard temperature, and pressure, dry (STPD) for $\dot{v}$O_2_ and $\dot{v}$CO_2_ calculations.

### Statistics

Application of the Bland–Altman Plot was used for the interpretation of comparisons between both Vyntus metabolic carts and both metabolic simulators. Differences between the VM and RT simulators, for different levels of BF for VT and RER and different levels of gas flow outcome values for VO_2_ and VCO_2_, were assessed using two-way analysis of variance (ANOVA). For analysis of bias, differences between metabolic simulator (VM, RT) vs. Vyntus (CPX, ONE) was tested by paired *t*-test. All data were analyzed using GraphPad Prism 5 (GraphPad Software, San Diego, CA 92108). Non-parametric Kendall’s Tau was used to check for correlation between proportional error of measured gas exchange estimates between metabolic simulators and Vyntus CPET systems (IBM Corp. Released 2020. IBM SPSS Statistics for Windows, Version 27.0. Armonk, NY: IBM Corp.). Significance was set at *p* < 0.05. Pre-defined acceptability limits (American Thoracic Society, 2003) are shown in Table 1.

**TABLE 1:** Tolerance of differences between metabolic simulator and metabolic cart.

**Breath frequency**	**VT (mL)**	**VT 2 L acceptability**	**VT (mL)**	**VT 3 L acceptability**	**$\dot{v}$O_2_; $\dot{v}$CO_2_**	**$\dot{v}$O_2_; $\dot{v}$CO_2_ acceptability**	**RER (–)**
**(/min)**		**range (mL)**		**range (mL)**	**(mL/min)**	**range (mL/min)**	
20	2,000	50	3,000	60	1,000	50	0.04
40	2,000	50	3,000	60	2,000	85	0.04
60	2,000	50	3,000	60	3,000	115	0.04
80	2,000	50	3,000	60	4,000	150	0.04

*VT, Tidal volume; $\dot{v}$O_2_, oxygen consumption; $\dot{v}$CO_2_, carbon dioxide production; RER, respiratory exchange ratio.*

## Results

### Tidal Volume (VT)

**FIGURE 2:**
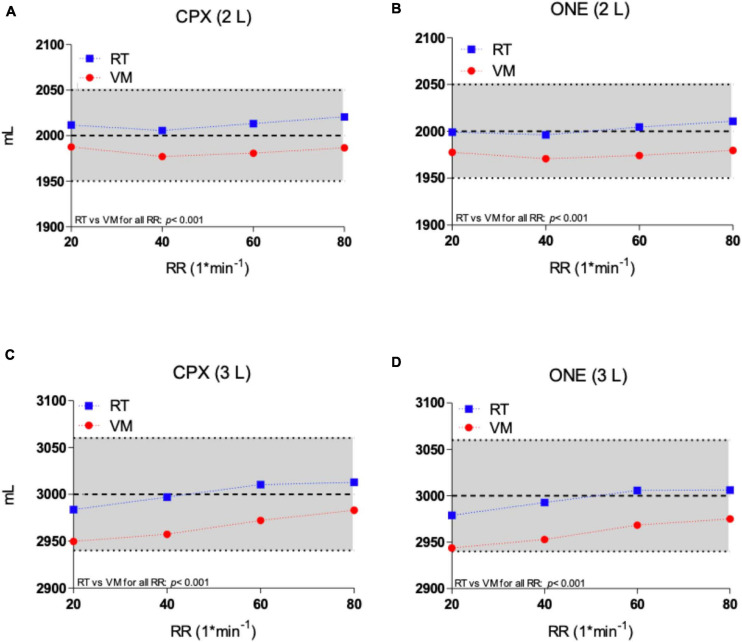
Tidal volume of Vacumed vs. Relitech simulators as measured by Vyntus CPX at 2 L **(A)** and 3 L **(C)** and Vyntus ONE at 2 L **(B)** and 3 L **(D)**. Each data point represents the mean ± SD of three sets of 40 breaths. VM, Vacumed; RT, Relitech. All data points *p* < 0.001; VM vs. RT.

Tidal volume results from the two simulators as measured by the Vyntus ONE and Vyntus CPX metabolic carts are shown in Figure 2. Mean VT results were significantly closer to the set point for the Relitech than for the Vacumed, but all results for both simulators were within acceptability ranges.

### Gas Exchange: Oxygen Uptake ($\dot{v}$O_2_), Carbon Dioxide Output ($\dot{v}$CO_2_)

**FIGURE 3:**
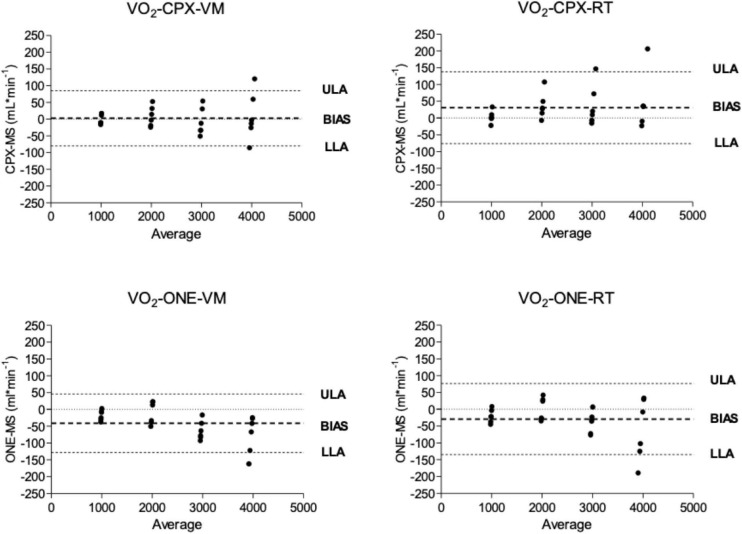
Bland and Altmann plots showing the difference between oxygen delivery (VO_2_) from metabolic simulator as measured by Vyntus devices. CPX, Vyntus CPX; VM, Vacumed metabolic simulator; RT, Relitech metabolic simulator; ONE, Vyntus ONE; ULA, Upper limit of agreement; LLA, Lower limit of agreement.

**FIGURE 4:**
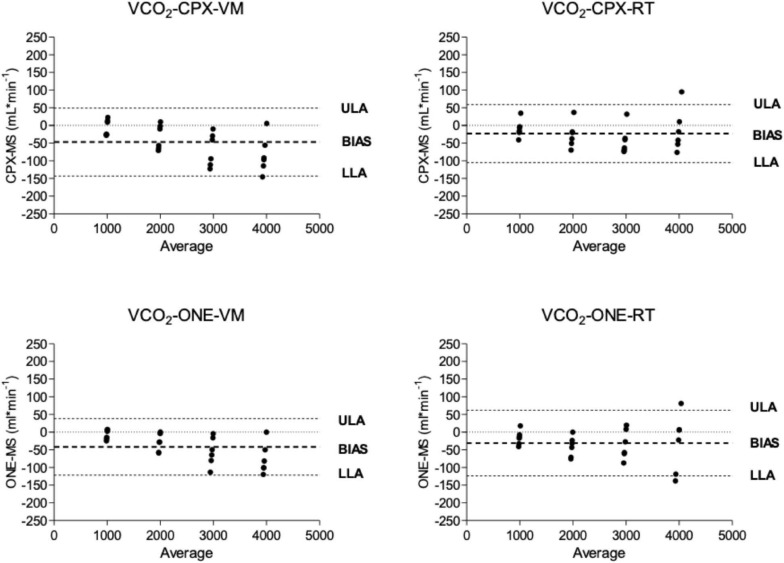
Bland and Altmann plots showing the difference between carbon dioxide delivery (VCO_2_) from metabolic simulator as measured by Vyntus devices. CPX, Vyntus CPX; VM, Vacumed metabolic simulator; RT, Relitech metabolic simulator; ONE, Vyntus ONE; ULA, Upper limit of agreement, LLA, Lower limit of agreement.

Oxygen uptake and carbon dioxide output flow rates as measured by Vyntus CPX and Vyntus ONE are shown in the Bland-Altman plots in Figures 3, 4. Although not all individual data points fell between the tolerance ranges for both simulators using both CPET devices, the mean bias for $\dot{v}$O_2_ and $\dot{v}$CO_2_ was less than 50 ml^∗^min^–1^ and the upper and lower limits of agreement are within the acceptability limits identified in Table 1. Mean and SD data supporting these plots is given in Table 2.

**TABLE 2:** Absolute gas ($\dot{v}$O_2_ and $\dot{v}$CO_2_) flow results for VT 2,000–3,000 mL.

			**Vyntus CPX**			**Vyntus ONE**		
(ml*min^–^**^1^)**	**VT**	**Simulator**	**VM mean ± sd**	**RT mean ± SD**	***p***	**VM mean ± SD**	**RT mean ± SD**	***p***
$\dot{v}$O_2_	2 L	1,000	1,015 ± 3	1,014 ± 18	*ns*	996 ± 6	988 ± 17	*ns*
		2,000	2,033 ± 19	2,062 ± 41	*ns*	2,020 ± 6	2,032 ± 9	*ns*
		3,000	3,024 ± 34	3,068 ± 81	*ns*	2,960 ± 24	2,983 ± 22	*ns*
		4,000	4,035 ± 74	4,077 ± 144	*ns*	3,956 ± 21	4,019 ± 23	**
	3 L	1,000	987 ± 3	994 ± 14	*ns*	969 ± 6	972 ± 22	*ns*
		2,000	1,965 ± 11	2,012 ± 17	*ns*	1,960 ± 9	1,971 ± 5	*ns*
		3,000	2,962 ± 10	3,008 ± 14	*ns*	2,916 ± 8	2,941 ± 26	*ns*
		4,000	3,983 ± 73	4,016 ± 34	*ns*	3,897 ± 69	3,861 ± 45	*ns*

			**Vyntus CPX**			**Vyntus ONE**		

$\dot{v}$CO_2_	2 L	1,000	1,015 ± 7	1,008 ± 23	*ns*	1,005 ± 3	996 ± 19	*ns*
		2,000	1,999 ± 10	2,000 ± 32	*ns*	1,990 ± 15	1,975 ± 22	*ns*
		3,000	2,973 ± 16	2,977 ± 49	*ns*	2,972 ± 32	2,978 ± 39	*ns*
		4,000	3,951 ± 52	4,017 ± 74	*ns*	3,939 ± 54	4,021 ± 53	*
	3 L	1,000	974 ± 2	974 ± 14	*ns*	981 ± 5	973 ± 17	*ns*
		2,000	1,936 ± 7	1,947 ± 16	*ns*	1,952 ± 17	1,943 ± 29	*ns*
		3,000	2,891 ± 14	2,939 ±18	*	2,919 ± 32	2,953 ± 49	*ns*
		4,000	3,883 ± 27	3,955 ± 30	***	3,910 ± 36	3,917 ± 79	*ns*

*VT, Tidal volume; VM, Vacumed; RT, Relitech; $\dot{v}$O_2_, oxygen consumption; CPX, Vyntus CPX; ONE, Vyntus ONE; $\dot{v}$CO_2_, carbon dioxide production; *ns*, not significant. ^∗^*p* < 0.05; ^∗∗^*p* < 0.01; ^∗∗∗^*p* < 0.001.*

From the $\dot{v}$O_2_ and $\dot{v}$CO_2_ results (Table 2) by both metabolic simulators it is shown that, using the Vyntus ONE at a VT of 2 L (both for $\dot{v}$O_2_ and $\dot{v}$CO_2_), a small but significant difference was found at 4,000 ml^∗^min^–1^. The Vyntus CPX only showed a small but significant difference at a VT of 3 L at 3,000 ml^∗^min^–1^ and 4,000 ml^∗^min^–1^ for $\dot{v}$CO_2_.

Bias with 95% confidence intervals between Vyntus CPET systems and metabolic simulators is shown in Table 3. Kendall’s Tau correlation coefficient to check for proportional error of gas exchange estimates by Vyntus systems is shown in Table 4.

**TABLE 3:** Bias with 95% confidence intervals between Vyntus CPET systems and metabolic simulators.

**$\dot{v}$O_2_**	**Bias**	**95% CI**	***p-*value**
CPX-VM	3	−15 to 21	0.748
CPX-RT	31	8 to 54	0.010
ONE-VM	−41	−60 to −22	0.000
ONE-RT	−29	−52 to −6	0.014

**$\dot{v}$CO_2_**	**Bias**	**95% CI**	***p-*value**

CPX-VM	−47	−68 to −27	<0.0001
CPX-RT	−23	−41 to −5	0.013
ONE-VM	−42	−59 to −25	<0.0001
ONE-RT	−31	−51 to −11	0.004

*$\dot{v}$O_2_, oxygen uptake; $\dot{v}$CO_2_, carbon dioxide output; CPX, Vyntus CPX; ONE, Vyntus ONE; VM, Vacumed metabolic simulator; RT, Relitech metabolic simulator.*

**TABLE 4:** Kendall’s Tau correlation coefficient to check for proportional error of gas exchange estimates by Vyntus systems.

**$\dot{v}$O_2_**	**Kendall’s Tau**	***p-*value**
CPX-VM	−0.082	0.607
CPX-RT	0.156	0.328
ONE-VM	−0.434	0.006
ONE-RT	−0.164	0.304

**$\dot{v}$CO_2_**	**Kendall’s Tau**	***p-*value**

CPX-VM	−0.488	0.002
CPX-RT	−0.213	0.181
ONE-VM	−0.532	0.001
ONE-RT	−0.057	0.719

*$\dot{v}$O_2_, oxygen uptake; $\dot{v}$CO_2_, carbon dioxide output; CPX, Vyntus CPX; ONE, Vyntus ONE; VM, Vacumed metabolic simulator; RT, Relitech metabolic simulator.*

### Respiratory Exchange Ratio (RER)

**FIGURE 5:**
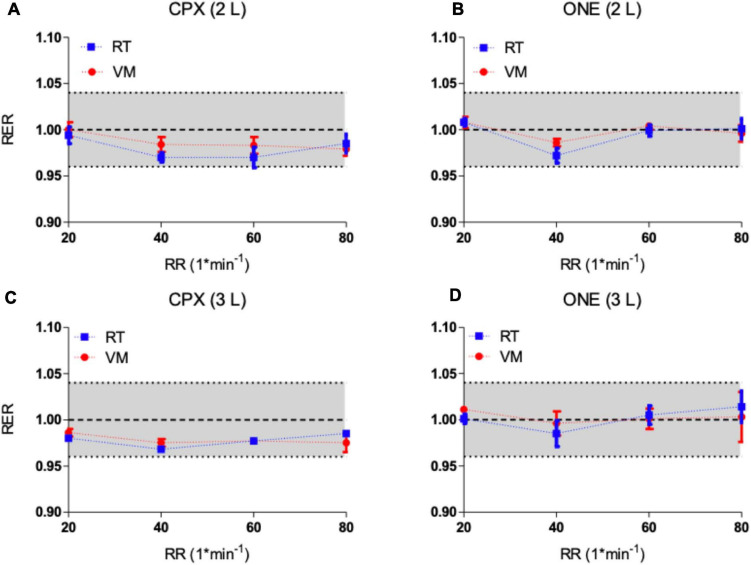
**(A,B)** Respiratory exchange ratio (RER) from metabolic simulators as measured by Vyntus CPX **(A)** and Vyntus ONE **(B)** at 2 L. **(C,D)** Respiratory exchange ratio (RER) from metabolic simulators as measured by Vyntus CPX **(C)** and Vyntus ONE **(D)** at 3 L. Data points represent the mean value ± SD of three sets of 40 breaths (simulator strokes). RT, Relitech; VM, Vacumed; RR, respiratory rate. All not significant.

Results of the respiratory exchange ratio ($\dot{v}$CO_2_/$\dot{v}$O_2_) from the two simulators as measured by Vyntus ONE and Vyntus CPX metabolic carts are shown in Figure 5. The results show that RER at all breathing frequency levels generated by both metabolic simulators were within acceptable ranges. Graphs indicate the mean ± SD for three sets of 40 breaths.

## Discussion

We compared the results of two state-of-the-art metabolic simulators using two different metabolic carts. We measured tidal volumes, respiratory exchange ratios, and oxygen and carbon dioxide mixtures at both 2 and 3 L stroke volumes of the metabolic simulators, over a range of respiratory rates from 20 to 80^∗^min^–1^.

Both the metabolic simulator and the metabolic cart have error tolerances within which they must operate. Metabolic cart tolerances are defined by the manufacturer-based design standards set for the device. In this study, tolerances for the Vyntus CPX and Vyntus ONE, as specified by the manufacturer, are VT ± 2% or 50 mL, $\dot{v}$O_2_ and $\dot{v}$CO_2_ ± 3% or 50 mL/min (whichever is greater), and RER difference = 0.04 absolute. Both metabolic simulators, based on the manufacturers’ calibration and certification reports, generate VT with a precision of 0.2%. $\dot{v}$O_2_ and $\dot{v}$CO_2_ were generated using high precision mass flow controllers by injection of pure (100%) N_2_ and CO_2_, resulting in a level of precision of <0.5% for $\dot{v}$O_2_ and $\dot{v}$CO_2_. Using these levels of tolerance, the values for acceptability ranges in Table 1 were derived.

Vyntus calibration procedures were followed as recommended by the manufacturer consisting of a volume and flow calibration for the digital volume transducer (DVT) and gas calibration procedure for O_2_ and CO_2_ gas analyzers. There are important differences in the calibration procedures for the two systems, a significant impetus for us to use two different metabolic simulators for each CPET cart in this study. For the Vyntus CPX, the DVT sensor is calibrated and verified by a flow signal, while the Vyntus ONE is manually calibrated and verified by an absolute volume.

The dynamic calibration procedure is designed to determine the gas sample delay and response time to align both gas sensor signals (O_2_ and CO_2_) with the corresponding ventilation as measured directly at the outlet of the mouthpiece/facemask by the DVT sensor. Gain is the ratio between actual and measured gas concentrations. Delay time is defined as the time needed for the gas to reach the analyzer via the sample line. Response time is the time needed between reaching the analyzer to showing the measured value (Porszasz et al., 2007). Knowing the total sum of delay and response time dynamically enables the system to set the calibration gas gain in relation to the O_2_ and CO_2_ sensor response. In a traditional non-dynamic gas calibration, without the exact estimate of the total of response and delay time, a difference between O_2_ and CO_2_ sensor response can lead to underestimation of gas concentrations at medium to high ventilation (breathing frequency) during exercise. At high ventilation, the end value of the sampled gas is typically not 100%. This concept also ensures the compensation of small fluctuations such as changes in the sample line performance over time. For our experiments, prior to each test, a duplicate gas/delay time procedure ensured correct and repeatable reading of the O_2_ and CO_2_ sensor aligned to breath-by-breath ventilation.

Through precise and accurate timing of the switching between room air and the calibration gas, values for gain, response time, and delay time for CO_2_ and O_2_ are determined. The switching frequency is equivalent to breathing rate (frequency) during CPET breath-by-breath measurements. Since CPET is a dynamic, breathing frequency-dependent procedure, gain factors determined in relation to breathing frequency will give the most accurate readings for both CO_2_ and O_2_ at any breathing frequency during metabolic exercise testing.

The SentrySuite Quality Management (SeSQM) software system provided the benefit of reading the CPET gas exchange outcome values under ambient environmental conditions as generated by the metabolic simulators. Normally gas exchange ($\dot{v}$O_2_/$\dot{v}$CO_2_) within a patient record is presented under standard temperature, pressure, and dry (STPD) conditions while ventilation values such as tidal volume are presented under body temperature, pressure, and water vapor saturated (BTPS). By using SESQM, no manual corrections of Vyntus data results were needed, enabling direct comparison of metabolic simulator results for this study.

As shown in Figure 2 and Tables 1, 2, VT was set at either 2 or 3 L with breathing frequencies of 20–80 breaths per minute. This corresponds to ventilation ranging from 40 to 240 L^∗^min^–1^. Both metabolic simulators generated stroke volumes within the predefined limits of acceptability. The VM model generated significant slightly lower volumes over the full range of frequencies compared to the RT simulator, but both were within acceptable ranges. No significant breathing frequency dependency was observed for a VT of 2 L. During the VT 3 L experiments, a minor frequency dependency could be observed by both metabolic simulators due to a low breathing frequency for CPET.

In Figures 3, 4, we have presented the measured valued generated by each metabolic simulator (RT and VM) in a Bland-Altman plot (Bland and Altman, 1986) presentation. The X-axes define the mean values from the simulators and the metabolic carts, while the Y-axes show the differences between the Vyntus measured results minus the metabolic simulator-generated output. A positive difference indicates a value measured by Vyntus that is higher than the value generated by the simulator and a negative value on the Y-axes indicates Vyntus result that is lower than that generated by the simulator.

Bland and Altmann graphs for both gas exchange parameters $\dot{v}$O_2_ and $\dot{v}$CO_2_ demonstrate that the variance of the difference between metabolic simulator-produced values increases with the increase of scale of the values. This is in accordance with the pre-set range of acceptability criteria varying from 50 ml^∗^min^–1^ for the lower values to 150 ml^∗^min^–1^ for the higher gas exchange values as shown in Table 1.

As shown by the Bland and Altman plots and the data in Table 2, repeated single results, even within a single metabolic simulator, show a range of variance. Based on these study results, it can be suggested that when a metabolic simulator is used, multiple measurements should be obtained for clinical quality control and decision-making. In our example, we used the mean of three sets of measurements.

Both Vyntus devices showed small but significant differences for $\dot{v}$O_2_ and $\dot{v}$CO_2_ as delivered by both simulators, except for $\dot{v}$O_2_ as delivered by the Vacumed simulator. However, all 95% confidence intervals stayed within the level of clinical acceptance. In addition, we found a relation between proportional error and measured estimates (in the range 1,000–4,000 mL^∗^min ^–1^) for Vyntus ONE for $\dot{v}$O_2_ and $\dot{v}$CO_2_ and for Vyntus CPX for VO_2_ when each device was connected to the Vacumed simulator. This is consistent with the expectations as reflected by an increased range of acceptance at higher measured gas flow estimates.

Because the RER is calculated from $\dot{v}$O_2_ and $\dot{v}$CO_2_, the negative difference of RER trend results from these combined effects (Figure 5). Although this tendency is apparent, the differences are still well within the defined range of acceptability.

The four chosen metabolic rates produced by the metabolic simulators, 1, 2, 3, and 4 liters per minute for $\dot{v}$O_2_ and $\dot{v}$CO_2_, corresponded to increasing the breathing frequency through 20, 40, 60, and 80^∗^min^–1^, respectively. In Figure 2, with the simulator’s VT set to 3 L, we can see that there is a minor difference between the simulator and Vyntus ONE at lower breathing rates (20 and 40^∗^min^–1^), and the difference increases with higher breathing rates (at 60 and 80^∗^min^–1^), proportional to the frequency. This can be observed in both $\dot{v}$O_2_ and $\dot{v}$CO_2_ results from Vyntus ONE.

The respiratory exchange ratio (RER) is an important parameter used in the interpretation of exercise testing results. For example, an RER over 1.05 indicates that the subject is close to peak exercise and the highest obtained RER during CPET is used in interpretation of exercise performance. Since RER is a derived parameter from $\dot{v}$O_2_ and $\dot{v}$CO_2_, the acceptability range is set at an absolute difference of 0.04 from the expected generated value of 1.00. As shown in Figure 5, the differences as measured by Vyntus CPX and Vyntus ONE were within the 0.04 limit of acceptability for the total range of frequency and metabolic states (breathing frequency, $\dot{v}$O_2_, and $\dot{v}$CO_2_). Strict criteria for the RER help to understand the balance between generated $\dot{v}$O_2_ and $\dot{v}$CO_2_. If $\dot{v}$CO_2_ is high (within the acceptable ranges) but $\dot{v}$O_2_ is low (within the acceptable ranges), a falsely high RER could be seen, thus potentially leading to misinterpretation of the CPET results of the subject tested.

Of note, RER is not influenced by variations in VT, as VT equally affects $\dot{v}$O_2_ and $\dot{v}$CO_2_. Because of this, RER relative to the absolute $\dot{v}$O_2_, $\dot{v}$CO_2_, and VT as generated by the simulator is a very powerful quality check of CPET analyzers.

## Conclusion

In our study, both standard metabolic simulators created comparable gas mixtures for O_2_ and CO_2_, to simulate highly accurate metabolic breath-by-breath parameters, within the ranges of pre-defined acceptability limits. The simulators are reliable across the spectrum of respiratory rates at tidal volumes of 2 and 3 L. Use of either of these metabolic simulators for standardized verification of metabolic cart equipment ensures accurate physiologic measurement of subjects undergoing cardiopulmonary exercise testing. No systematic differences were identified when comparing the two types of simulators.

Despite the use of a metabolic simulator, CPET labs must ensure the proper performance of a metabolic cart using both biological control (repeated measurements of a human subject over time) and expert clinical quality control with clinical interpretation of normal human subject testing.

## Data Availability Statement

The original contributions presented in the study are included in the article/supplementary material, further inquiries can be directed to the corresponding author/s.

## Author Contributions

TS and HG were involved equally in the study design, data acquisition, interpretation of data, and review of the manuscript. ER was involved in the interpretation of data and wrote the manuscript. All authors contributed to the article and approved the submitted version.

## Conflict of Interest

TS and ER are paid consultants for Vyaire Medical, Mettawa, IL, United States. HG is an employee of Vyaire Medical, Mettawa, IL, United States. Financial support for this project came from Vyaire Medical, Mettawa, IL, United States.
